# Genome-wide association study reveals the genetic basis of rice resistance to three herbicides

**DOI:** 10.3389/fpls.2024.1476829

**Published:** 2024-10-01

**Authors:** Peizhou Xu, Yuhe Qin, Maosen Ma, Tengfei Liu, Fenhua Ruan, Le Xue, Jiying Cao, Guizong Xiao, Yun Chen, Hongyan Fu, Gege Zhou, Yonghua Xie, Duo Xia

**Affiliations:** ^1^ State Key Laboratory of Crop Gene Exploration and Utilization in Southwest China, Rice Research Institute, Sichuan Agricultural University, Chengdu, China; ^2^ Department of Research and Development, Luzhou Taifeng Seed Industry Co., Ltd., Luzhou, Sichuan, China; ^3^ Department of Research and Development, Zoeve Seed Co., Ltd., Chengdu, Sichuan, China

**Keywords:** rice, herbicide resistance, genome-wide association studies, genetic diversity, glufosinate, glyphosate, mesotrione

## Abstract

Crop resistance to herbicides is crucial for agricultural productivity and sustainability amidst escalating challenges of weed resistance. Uncovering herbicide resistant genes is particularly important for rice production. In this study, we tested the resistance to three commonly used herbicides: glufosinate, glyphosate and mesotrione of 421 diverse rice cultivars and employed genome-wide association studies (GWAS) to unravel the genetic underpinnings of resistance to these three herbicides in rice. We discovered that cultivated rice exhibited rich variation in resistance to the three herbicides, and the differences among subpopulations were significant. Six identified associations harboring candidate genes for resistance to these herbicides were significant. Among them, *RGlu6* and *RGly8* were the major QTL for resistance to glufosinate and glyphosate, respectively. The favorable alleles of *RGlu6* and *RGly8* were primarily present in *japonica* cultivars that originated from Europe, highlighting the geographic and genetic diversity of herbicide resistance and emphasizing the localized selection pressures in European rice varieties. Moreover, our findings might suggest that traditional target genes may not contain tolerant alleles in nature, and alternative mechanisms with novel loci associated with resistance may work. By mapping the genes for herbicide resistance, our results may help develop new strategies to combat the dual challenges on effective weed management and herbicide sustainability.

## Introduction

Rice is a crucial staple food for more than half of the global population, playing an essential role in agricultural production, especially in Asian countries (FAO, http://www.fao.org). Weeds pose a horrible threat to rice by competing for nutrients, water, sunlight, and space, and by increasing the incidence of diseases and insect pests (Akbar et al., 2011). This competition can lead to substantial reductions in rice yield and grain quality, up to over 40% due to weed damage (Burgos et al., 2014). The shift from transplanting to direct seeding, driven by urbanization and labor shortages has exacerbated weed problems (Chaudhary et al., 2023). Traditional methods for weed control, such as tillage, irrigation, hand-weeding etc., are labor-intensive and resource-demanding. Using herbicides is the most effective way to control weeds, but many herbicides also damage or kill rice plants (Jin et al., 2022). The development and use of herbicide-resistant cultivars for an effective control of weeds without harming the plant have therefore become critical in modern agricultural practices (Kawahara et al., 2013).

Glufosinate, glyphosate and mesotrione are three widely used herbicides and significant progress has been made in understanding and developing resistant plants to these herbicides. As a major competitive inhibit target of glufosinate, glutamine synthase (GS) plays a pivotal role in plants’ resistance to glufosinate and mutations in GS genes could enhance plants’ resistance to glufosinate (Tian et al., 2015). Glyphosate is a broad-spectrum post-emergent herbicide and mainly targets 5-enolpyruvylshikimate-3-phosphate synthase (EPSPS), a key enzyme in the biosynthesis of aromatic amino acids, and phenolics, and gain of function of EPSPS genes could endow plants with resistance to glyphosate (Sammons and Gaines, 2014). It has been reported that acetyltransferases (ACEs) synthesizing genes can be induced by mesotrione, indicating a role for ACEs in resistance to mesotrione of crops (Chen et al., 2022). In addition, other genes like *OsACC1* which encodes acetyl-CoA carboxylase and *OsALS1* which encodes acetolactate synthase were reported to be responsible for resistance to herbicides like aryloxyphenoxypropionates (APPs) and pyrimidinyl carboxy (PC) herbicides, respectively (Okuzaki et al., 2007; Xu et al., 2021). Researchers have successfully created herbicide-resistant rice varieties through artificial mutagenesis, genome editing, introducing and overexpression of these genes (Li et al., 2016; Maeda et al., 2019; Chen et al., 2021; Ren et al., 2023; Zhang et al., 2023). Despite these advancements, the genetic resources for herbicide resistance are still limited in rice. The evolution of herbicide-resistant weeds due to the extensive use of chemical control strategies has further complicated this issue. For instance, over 255 weed species worldwide have developed resistance to at least one herbicide, including 42 species resistant to glyphosate (Perotti et al., 2019). This situation underscores the need for continuous research and development of new herbicide resistance mechanisms and the identification of novel resistance genes to ensure sustainable rice production.

Genome-wide association studies (GWAS) have emerged as a powerful tool for dissecting the genetic basis of complex quantitative traits in plants (Zhou et al., 2017, 2021a, 2021b). This method enables the identification of genetic variants associated with specific traits by analyzing the entire genome (Xia et al., 2022). In this study, we employed GWAS to investigate the genetic basis of resistance to three different herbicides in a diverse rice germplasm population. This approach allowed us to identify key genetic loci associated with herbicide resistance, providing valuable insights into the development of new herbicide-tolerant rice varieties. By leveraging the genetic diversity present in natural populations, GWAS offers a robust framework for enhancing our understanding of herbicide resistance and for breeding rice varieties with improved resistance profiles.

## Materials and methods

### Plant materials and phenotyping

The 421 cultivated rice varieties used in this study were selected from diverse germplasms collected domestically and internationally, and the names, geographical origin, and subpopulation information of which have been previously reported (Zhao et al., 2015). In 2022, these rice varieties were planted at the South Breeding Base of Sichuan Agricultural University in Lingshui County, Hainan Province, China. The seeds of each were individually harvested in April 2023, uniformly air-dried and placed in the seed storage facility in the Rice Research Institute of Sichuan Agricultural University in Chengdu, Sichuan Province.

Sixty grains of each accession were sown in a flowerpot (15 cm diameter and 16 cm height) in July 2023 with three replications for herbicide evaluation in the Chengdu Campus of Sichuan Agricultural University. Half-lethal dose of glyphosate, glufosinate and mesotrione solutions was sprayed, respectively at the 3-4 leaf stage as 2.25 L/hm^2^ of 41% glyphosate (Fuhua Chemical), 3.00 L/hm^2^ of 20% glufosinate (BASF) and 1.20 L/hm^2^ of 10% mesotrione (Hubei Jiahui Xingcheng Biotechnology Co., Ltd.). The response of each pot to herbicide injury was scored with 0-4 scale five days after the application of glyphosate and glufosinate, but seven days after the application of mesotrione, respectively, where 0 was for the plants with no apparent injury, level 1 for slight effect on seedlings, level 2 for moderate effect, level 3 for severe effect and 4 with the most severe injury.

### Genome wide association study

GWAS analyses for herbicide resistance traits were performed for the entire panel and for *indica* and *japonica* group separately, using mixed linear models to take population structure and relative kinship into consideration for statistical association. SNPs with a minor allele frequency (MAF) greater than 5% and a missing rate less than 15% were selected for the association analysis, and 6.3 million variants, including 5.5 million SNPs and 800,000 InDels, were used for GWAS, as well as estimating population structure and kinship coefficients. The thresholds of genome-wide significance were determined using a modified Bonferroni correction as described by Li et al. (2012), where the total number of SNPs (M) used for threshold calculation was replaced by the effective number of SNPs (Me), in mixed linear models provided by EMMAX program (Kang et al., 2010). The physical locations of the SNPs and InDels were identified based on the Rice Annotation version 7.0 of the variety Nipponbare (Kawahara et al., 2013) from the website http://rice.uga.edu/.

The Bayesian clustering program fast Structure (Raj et al., 2014) was used to calculate different levels of *K* (*K* = 2-5), and the command choose_K.py was employed to identify the model complexity for maximizing the marginal likelihood. LD heatmaps and gene haplotypes were constructed using SNPs and InDels (MAF >0.03) from the 1 Mb regions around each peak SNP using R package ‘LD heatmap’ (Shin et al., 2006).

### Variant-function-based association analyses

We annotated the functional variations within a 1 Mb region surrounding the associated loci using snpEff software (Cingolani et al., 2012), and classified all polymorphisms in the candidate region into four groups, as for 1) those predicted to induce amino acid exchanges or to change splicing junctions (GT or AG at the beginning or end of an intron, respectively), 2) those located at the 5′ flanking sequences of genes (e.g., within 2kb of the first ATG, typically the promoter region), 3) those located within a gene but did not meet the criteria for Group I or II (for example, located in a coding region but not predicted to change an amino acid, an intron, or a 3′ noncoding sequence), and 4) those located outside of coding regions. By identifying the most significant variant in Group I, potential candidate genes were determined.

### Gene haplotype and geographic distribution analyses

The haplotype of each candidate gene was analyzed by downloading the genotypes of variant sites located 2kb upstream of the transcription start site and within the coding sequence of the gene from the RiceVarMap 2.0 (ricevarmap.ncpgr.cn) (Zhao et al., 2021). To improve the efficiency of the haplotype analysis, those variant sites with a minor allele frequency less than 0.05 and a missing rate greater than 10% were filtered out. The genotypes of remaining high-quality variants were used for haplotype classification. To better display the distribution pattern of haplotypes, only those haplotypes containing more than 10 varieties were shown. The information on geographical distribution of the 421 accessions was also downloaded from the RiceVarMap website. The world map was plotted using the ‘maps’ package in R (Becker and Wilks, 1995), and the characteristics of different haplotypes on geographical distribution were displayed in pie chart form using the ‘ape’ package in R (Paradis and Schliep, 2019).

### Statistical analyses

Violin plots, correlations, and multiple comparisons were constructed using phenotypic means from three replicates for each accession. The *P* values for Pearson’s correlation coefficients were calculated by two-sided *t*-tests using the cor.test() function in R (Ihaka and Gentleman, 1996). Multiple comparisons of phenotypic data among different subgroups or haplotypes were conducted using the duncan.test() command in the ‘agricolae’ package in R, with a significant probability at 0.01. The ‘vioplot’ package in R was used to create violin plots for the multiple comparisons (https://github.com/TomKellyGenetics/vioplot).

## Result

### The distribution of three herbicide resistance in rice germplasm

**Figure 1:**
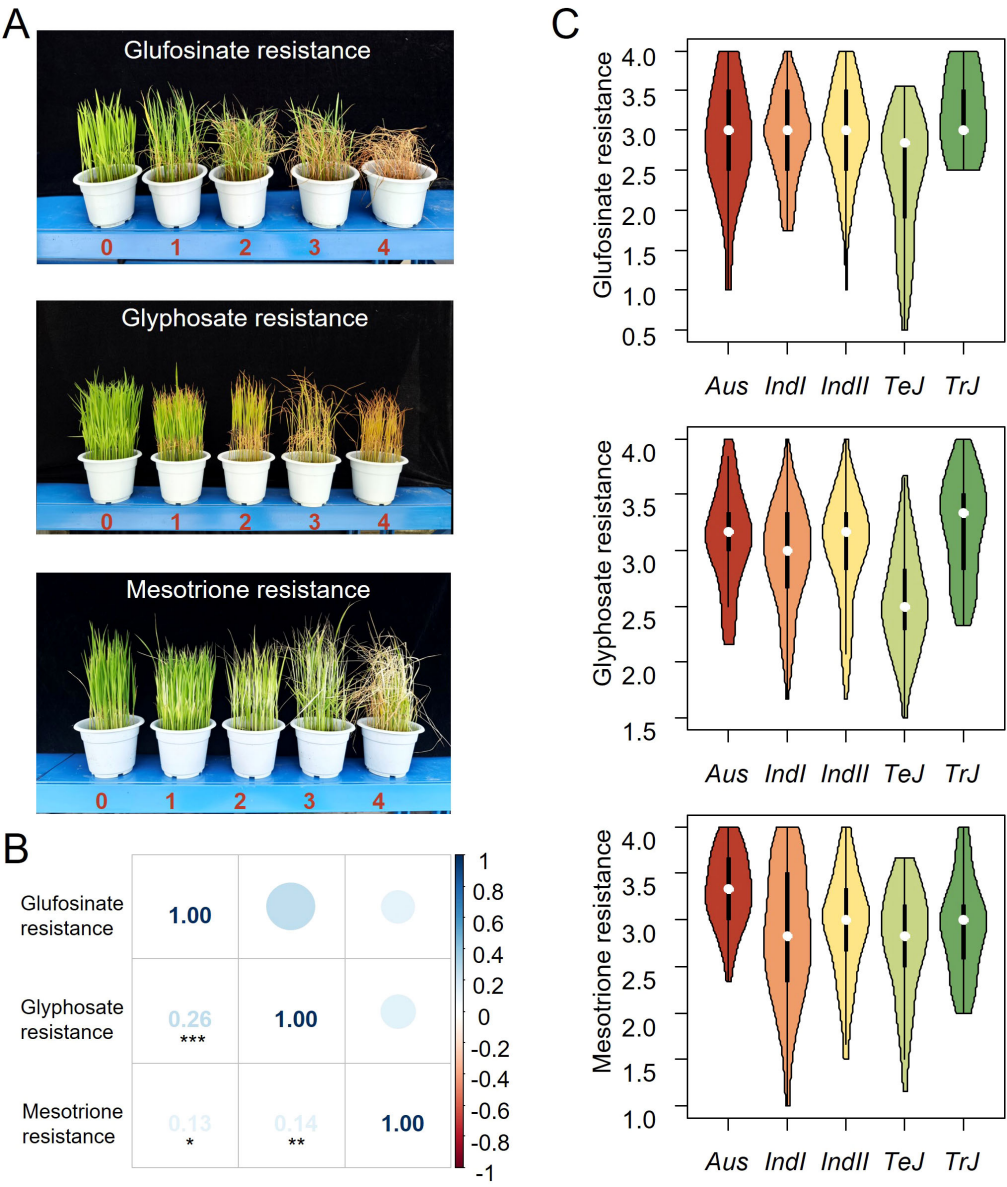
Identification, phenotypic distribution, and correlation of resistance to three herbicides in cultivated rice. **(A)** Grading criteria for tolerance to three herbicides. **(B)** Correlation among three herbicide tolerances in cultivated rice. **(C)** Phenotypic distribution of tolerance to three herbicides across different subgroups in cultivated rice.

By grading the herbicide resistance, we observed significant differences among the 421 accessions responding to the three herbicides, indicating the feasibility of the grading standards (**Figures 1A**; **Supplementary Figure S1**). The cultivars generally exhibited low resistance to all three herbicides, with average resistance ratings around level 3. Less than 5% cultivars exhibited relatively high resistance (resistance ratings smaller than level 2 to the three herbicides, and a *Temperate Japonica* cultivar HB-6-2 was the relatively highest glufosinate and glyphosate resistant cultivar in the tested materials (**Supplementary Figure S1**; **Supplementary Tables S4**, **S5**). There is a significant positive correlation between the resistance to the three herbicides in cultivated rice (*r^2 ^= *0.26,0.13,0.14), although the correlation coefficients are relatively low (**Figure 1B**). Among them, the positive correlation between glufosinate resistance and glyphosate resistance is the most significant, with a correlation coefficient of 0.26 (*p*= 7.5 × 10^-5^).

### Different subpopulations varied in resistance to the three herbicides

A total of 6.3 million high-quality variants, including 5.5 million SNPs and 0.8 million InDels, queried from RiceVarMap (http://ricearmap.ncpgr.cn), were used to estimate population structure and *Kinship* coefficients, as well as GWAS. When *K* equaled 2, the 421 accessions in the whole panel were divided into 300 *Indica*, 106 *Japonica* and 15 *Intermediate* ones. And When *K* equaled 5, the 300 *Indic*a accessions were further divided into 38 *Aus*, 143 *Indica I* (*IndI*), 119 *Indica II* (*Ind II*), while the 106 *Japonica* accessions were divided into 60 *Temperate Japonica* (*TeJ*) and 46 *Tropical Japonica* (*TrJ*). Based on this population structure, we noticed a varying resistance to different herbicides of different subgroups (**Figure 1C**). For glufosinate and glyphosate, the *TeJ* subgroup exhibited significantly higher resistance than other subgroups (**Figure 1C**); for halosulfuron-methyl, the *Aus* subgroup showed significantly lower resistance than other subgroups. Thus, the cultivars with different genetic backgrounds demonstrated diverse herbicide resistance.

### Loci associated with herbicide resistance

The strict inbreeding mating habits of rice have led to significant subpopulation structure and considerable linkage disequilibrium (LD), which reduce the mapping resolution of association studies and increase the occurrence of type I errors (Zhou et al., 2017). To address these issues, we used a mixed model to correct the interferences from subpopulation structure by incorporating the first three principal components (PCs) as covariates in the model, and conducted genome-wide association studies (GWAS) in the *Indica* group (303 accessions), *Japonica* group (181 accessions), and the entire group (all varieties) (see Methods). The calculated genome-wide significance thresholds, based on a nominal level of 0.05, were *P* = 6.6 × 10^-8^, 8.7 × 10^-8^, and 2.0 × 10^-7^ for the whole panel, *Indica* and *Japonica*, respectively (**Supplementary Table S1**).

**Figure 2:**
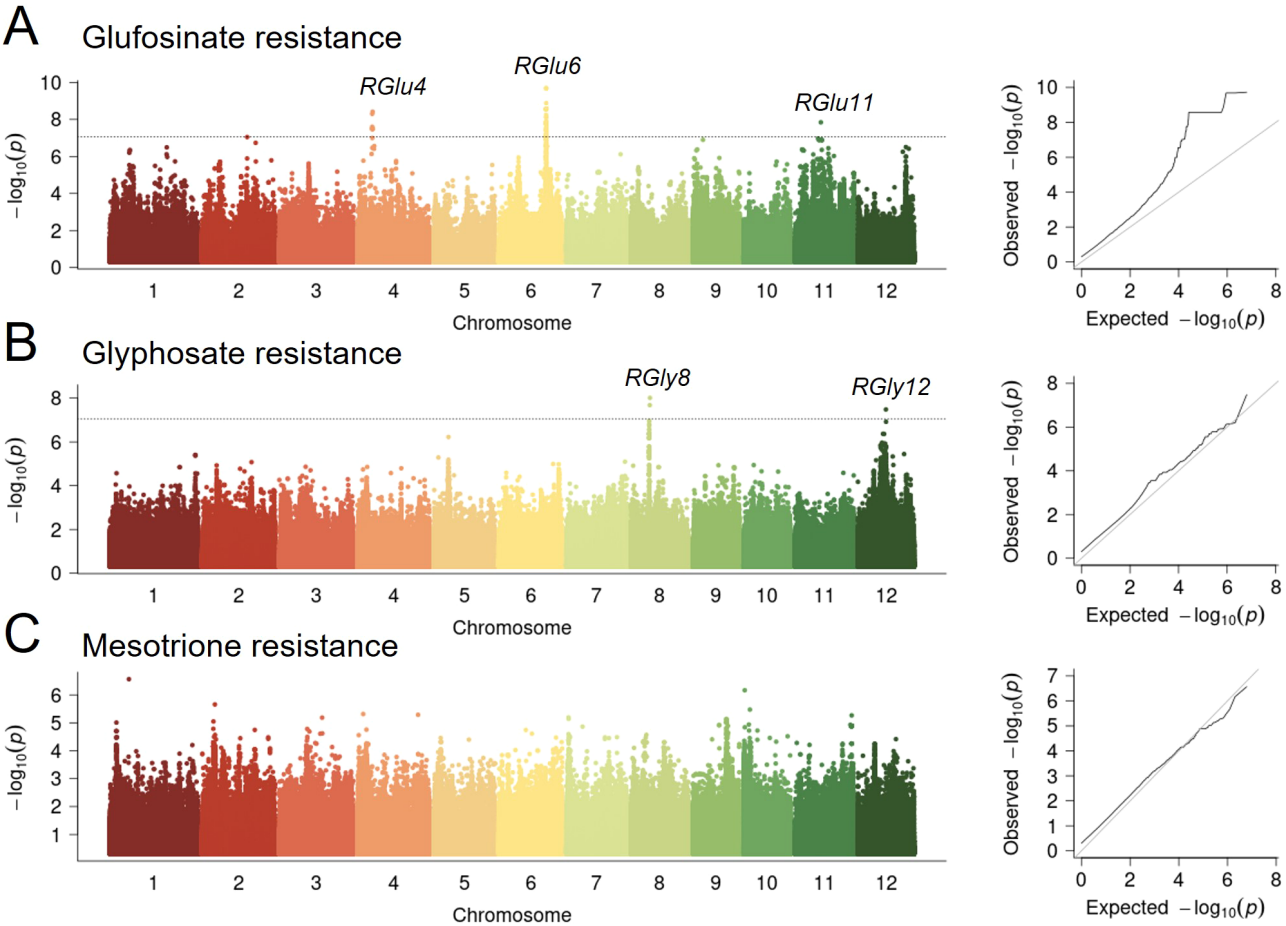
Genome-wide association results for resistance to three herbicides in 421 cultivated rice. Manhattan plots and quantile–quantile plots depicting GWAS results using a mixed model for tolerance to glufosinate **(A)**, glyphosate **(B)** and mesotrione **(C)**. Associations identified in all accessions (lower), indica subspecies (middle), and japonica subspecies (upper). The x axis depicts the physical location of SNPs across the 12 chromosomes of rice and the y axis depicts the -log_10_ (*P* value).

Five significant loci associated with resistance to glufosinate and glyphosate, but none to mesotrione were identified in the whole panel (**Figure 2**; **Supplementary Figures S2**, **S3**). Detailed association results for each herbicide in each subpopulation are presented in **Table 1**. Three loci associated with glufosinate resistance in the whole panel totally explained 25.76% of the phenotypic variance, with effect of each varying from 15.95% to 19.20% (**Supplementary Table S2**). For glyphosate resistance, two loci accounted for 26.69% of the phenotypic variance in total, and 5.15% and 20.06% alone, indicating a significant role of the additive effects on the variation of glyphosate resistance (**Supplementary Table S3**).

**Table 1:** SNPs and candidate genes significantly associated with herbicides resistance.

Trait	Allele^a^	Chr	Pos^b^	*P* value	QTL	Dis(kb)^c^	Candidate gene^d^	Description
Glufosinate	A/G	4	7431857	3.81E-09	*RGlu4*	-40	*LOC_Os04g13130*	hypothetical protein
Glufosinate	A/G	6	23190901	2.07E-10	*RGlu6*	6	*LOC_Os06g39070*	UDP-glucosyl transferase
Glufosinate	A/C	11	13551277	1.87E-08	*RGlu11*	9	*LOC_Os11g08190*	expressed protein
Glyphosate	A/G	8	8954806	1.11E-08	*RGly8*	-7	*LOC_Os08g14850*	resistance protein
Glyphosate	G/C	12	12637368	5.10E-08	*RGly12*	40	*LOC_Os12g22460*	hypothetical protein
Mesotrione	T/C	1	9366585	9.69E-07	*RMes1*	6	*LOC_Os01g16510*	expressed protein

a Major allele/minor allele; underlined base is the reference allele. Position in base pairs for the lead SNP according to the Nipponbare reference genome version 7. Distance from the candidate gene to the peak SNP. Plausible biological candidate gene in the locus or the nearest annotated gene to the lead SNP.

### Functional and haplotype analyses of *RGlu6* for rice glufosinate resistance

**Figure 3:**
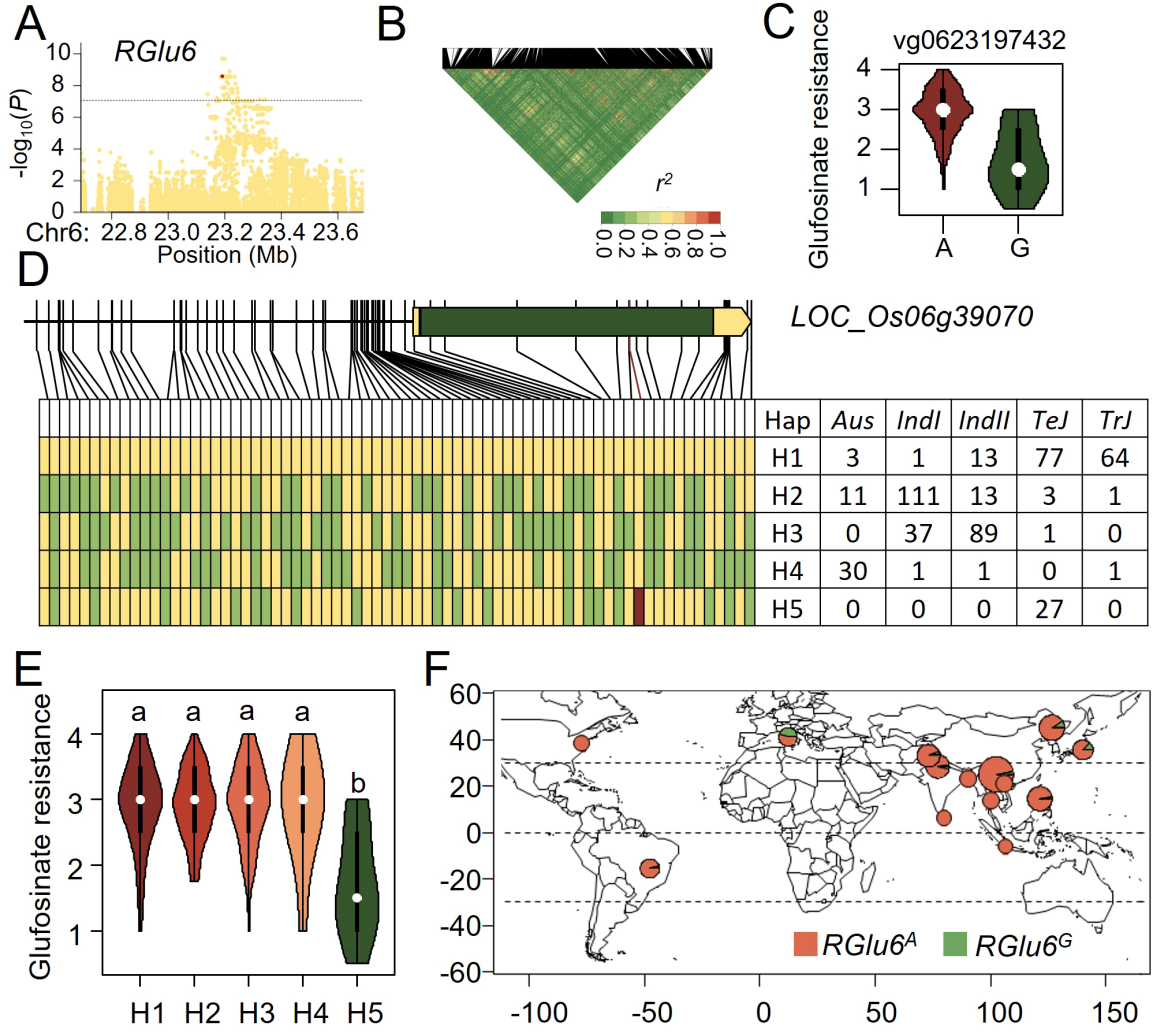
Natural variation of *RGlu6* affect glufosinate resistance in rice. **(A)** Manhattan plot for the 1 Mb region around the glufosinate resistance-associated locus *RGlu6*. **(B)** LD heatmap for the region around *RGlu6*. **(C)** Potential functional variants at *RGlu6* and their effects on glufosinate tolerance. **(D)** Haplotype analysis of the candidate gene at *RGlu6*. Yellow represents the reference genome genotype, green represents alternate genotypes, and red represents functional variants. **(E)** Phenotypic distribution of the five haplotypes at *RGlu6*. **(F)** Geographic distribution of accessions with different *RGlu6* allele in the GWAS panel.

Since *RGlu6* explained the highest phenotypic variation (*R^2 ^= *19.20%, **Supplementary Table S2**), we chose it as a major QTL for rice glufosinate resistance and conducted variant-function-based association analysis of the associated loci regions on *RGlu6* (see Methods). By analyzing the function of variants within 500 kb upstream and downstream of the peak SNP and the linkage disequilibrium (LD) blocks in that segment (**Figures 3A, B**), we analyzed the candidate genes and functional variants of *RGlu6*. LD decay around *RGlu6* was rapid with no obvious LD blocks, where the most significant functional variant (vg0623197432) was only 6 kb away from the peak SNP (**Table 1**). A significant glufosinate resistance difference between type A and type G of vg0623197432 variant was observed (*p*= 1.8×10^-10^, **Figure 3C**). Since variant vg0623197432 was located on the exon of *LOC_Os06g39070*, which encodes a UDP-glucosyl transferase (UGT), we consider it as a candidate gene for *RGlu6*.

Using the genotypes of the 2 kb promoter and coding region variations of *LOC_Os06g39070* with 61 SNPs and 13 InDels, we categorized *RGlu6* of these 421 cultivars into 5 haplotypes, namely H1 - H5 (**Figure 3D**). Haplotype H1 was predominantly present in *TeJ* and *TrJ*, H2 and H3 mainly in *IndI* and *IndII*, H4 in *Aus*, and H5 only in *TeJ*, respectively. Multiple comparison demonstrated no significant differences were among haplotypes H1-H4, while haplotype H5 exhibited significantly higher resistance to glufosinate than others, indicating beneficial allelic variations of H5 haplotype (**Figure 3E**). Thus, we classified *RGlu6* into two allelic genotypes with merging the indistinct H1-H4 into *RGlu^A^* genotype, and considering H5 as *RGlu^G^* genotype. By analyzing the geographic distribution of these allelic genotypes (**Supplementary Table S4**), we observed that the accessions with the *RGlu6^G^* genotype mainly originated from Europe (**Figure 3F**), which indicates that *RGlu6^G^* may have arisen from a mutation and was retained locally through natural selection.

### Functional and haplotype analyses of *RGly8* for rice glyphosate resistance

**Figure 4:**
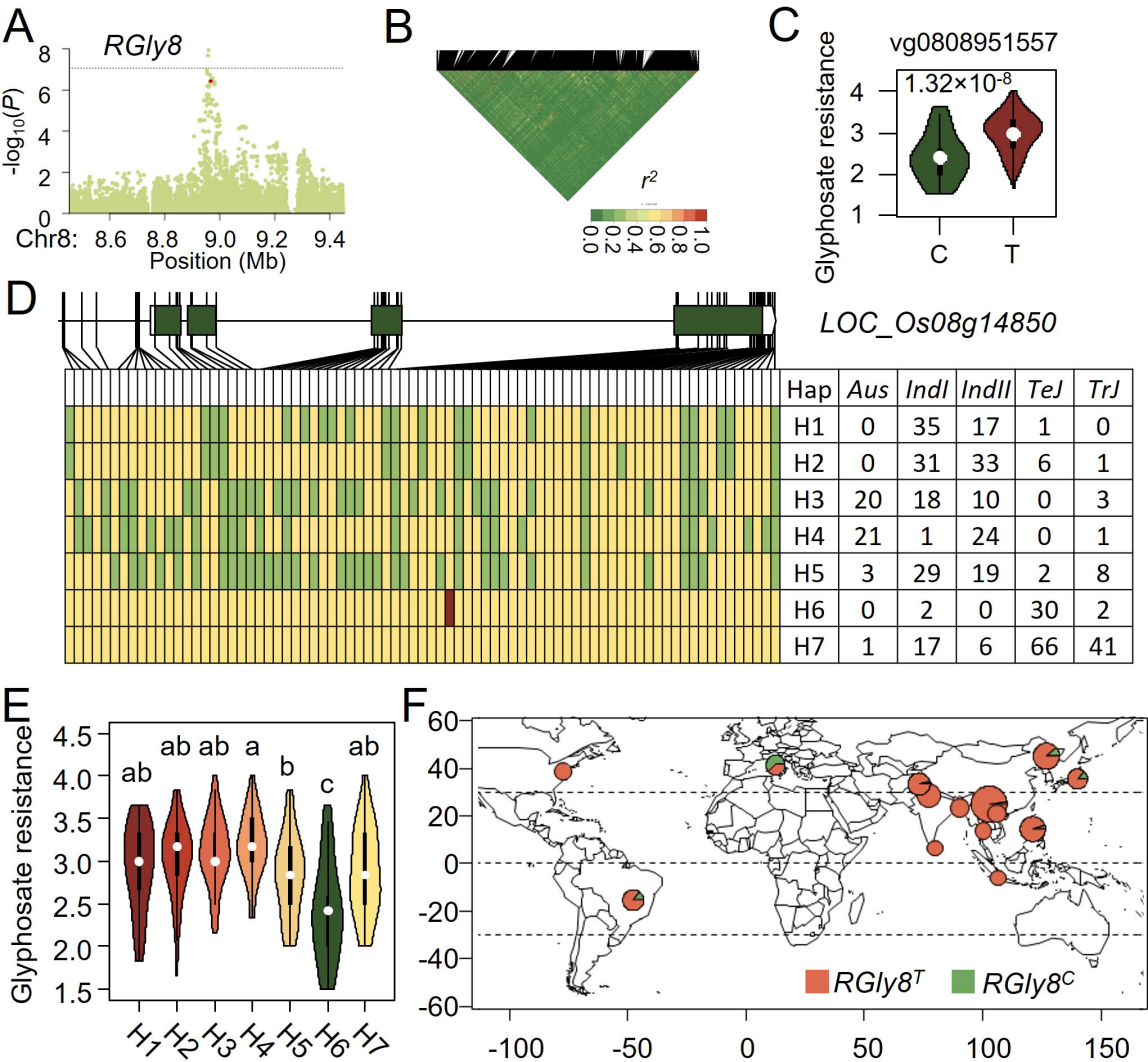
Natural variation of *RGly8* affect glyphosate resistance in rice. **(A)** Manhattan plot for the 1 Mb region around the glyphosate resistance-associated locus *RGly8*. **(B)** LD heatmap for the region around *RGly8*. **(C)** Potential functional variants at *RGlu6* and their effects on glyphosate tolerance. **(D)** Haplotype analysis of the candidate gene at *RGly8*. Yellow represents the reference genome genotype, green represents alternate genotypes, and red represents functional variants. **(E)** Phenotypic distribution of the five haplotypes at *RGly8*. **(F)** Geographic distribution of accessions with different *RGly8* allele in the GWAS panel.

Analyzing candidate genes at the *RGly8* locus for rice glyphosate resistance in a rapid LD decay around *RGly8*, we found that vg0808951557 was the most significant functional variant located only 7 kb from the peak SNP (**Figures 4A, B**). The T to C mutation of vg0808951557 which was in the fourth exon of *LOC_Os08g14850*, led to an amino acid changed from serine to proline (**Figure 4C**). Since *LOC_Os08g14850* encodes a resistance protein and was reported to be involved biotic and abiotic stresses, we considered it as a candidate gene for *RGly8*.

Seventy four SNPs and five InDels were used to analyze the haplotypes of *RGly8* and seven haplotypes, namely H1 - H7 were categorized based on the glyphosate resistance and genotypes of the 421 accessions (**Figure 4D**). Haplotypes H1-H5 were predominantly present in *Indica*, while H6 and H7 were mainly present in *Japonica*. The superior C genotype of vg0808951557 was exclusively found in the H6 haplotype. Multiple comparisons revealed significant differences for glyphosate resistance among these seven haplotypes, with H6 haplotype exhibiting a significantly higher glyphosate resistance than other haplotypes (**Figure 4E**). Therefore, we designated the H6 haplotype as the allelic genotype *RGly8^C^*, and the other haplotypes as *RGly8^T^*. Analysis of the geographic distribution of these alleles showed that the superior allele *RGly8^C^* primarily originated from Europe, the same as that of *RGlu6^G^* (**Figure 4F**; **Supplementary Table S4**).

These results suggested that resistance to both glufosinate and glyphosate may have been locally selected in Europe, leading to an increase in the frequency of superior resistance alleles in rice accessions originated from that region.

## Discussion

The intense application of herbicides has effectively assisted in weeds control but meanwhile accelerated the evolution of resistance to herbicides of both crops and weeds (Peterson et al., 2018). Discovering and developing herbicide-resistant crop is one of the most promising ways for weed control and crop production. In this study, we tested the resistance to three frequently herbicides, namely glufosinate, glyphosate and mesotrione, of 421 diverse rice accessions. Varying degrees of resistance to these herbicides were discovered (**Supplementary Figure S1**), indicating the possibility of natural herbicide-resistant rice varieties. Besides, a relatively higher resistance in average to these three herbicides in *TeJ* compared to other subpopulations were observed (**Figure 1C**). Since TeJ mainly originated from Europe, a higher herbicides selection pressure in Europe might be the cause (Peterson et al., 2018).

GS and EPSPS are the major competitively inhibited targets of glufosinate and glyphosate, respectively, and rice varieties endowed with resistance to these two herbicides were mostly genetically modified on genes encoding GS and EPSPS (Achary et al., 2020). *OsGS1;1* and *OsGS2*, encoding glutamine synthetase, were reported to be involved in rice growth and resistance to abiotic stresses, but the resistance to glufosinate has not been reported (Cai et al., 2010; James et al., 2018). Our study aimed to identify natural variant loci associated with resistance to either of glufosinate and glyphosate in rice through GWAS, but none was detected in their target gene regions (**Figures 2A, B**, **Supplementary Figures S2**, **S3**). This result suggested that either these target genes might not possess alleles to confer natural herbicide resistance, or such natural resistance alleles may be either too weak or too rare to be detected by GWAS.

Since the 421 geographically and genetically diverse rice cultivars displayed various resistance to the three herbicides (**Figure 1C**, **Supplementary Figure S1**) and QTL which were reported to be associated with resistance to these herbicides did not overlap with neither EPSPS genes nor GS genes (Bao et al., 2022; Xu et al., 2023), alternative mechanisms of plant herbicide resistance beyond just reducing the direct interaction between the target gene protein and the herbicide might exist. In the association analysis for glufosinate resistance, we defined *LOC_Os06g39070* which encodes a UDP-glucosyl transferase (UGT) as a candidate gene. UGT is a class of enzymes widely found in plants, whose primary function is to transfer glucose units from uridine diphosphate glucose (UDP-glucose) to a variety of acceptor molecules, including hormones, toxins, and secondary metabolites (Lairson et al., 2008; Tiwari et al., 2016). In rice, UGT genes participates in response to abiotic and biotic stresses, and gain of function of OsUGTs could improve rice resistance to various stresses through elevating scavenging reactive oxygen species (ROS) and plant hormones (Wang et al., 2021, 2023; He et al., 2023). Thus, *LOC_Os06g39070* might reduce the toxicity of glufosinate through hormones or ROS scavenging processes. In the association analysis for glyphosate resistance, *LOC_Os08g14850* which encodes resistance protein CC-NBS-LRR was detected (**Figures 2B**, **4**). Previous studies have shown that resistance proteins play a role not only in plant responses to biotic stress but also in responses to abiotic stress (Atkinson and Urwin, 2012). *LOC_Os08g14850*/*SCR8* has been reported to be involved in rice’s resistance to bacterial blight and mutant *scr8* displayed a defected root growth phenotype (Hu et al., 2021). Taking these together, *LOC_Os08g14850* might be a potential target for enhancing rice resistance to glyphosate.

Haplotype analysis of *RGlu6* and *RGly8* revealed that superior alleles of *RGlu6* and *RGly8* are predominantly present in *TeJ* (**Figures 3D, E**, **4D, E**). Geographic analysis of *RGlu6^G^* and *RGly^C^* showed that either of them were originated from Europe (**Figures 3F**, **4F**). These results indicated a possible severe herbicide pressure in Europe (Devos et al., 2006).

In summary, this study reveals the genetic basis of rice resistance to three herbicides through genome-wide association analysis, offering new insights for deciphering new mechanisms of herbicide resistance and breeding herbicide-resistant rice varieties.

## Conclusion

Enhancing rice resistance to herbicides is the most effective way to maintain rice production against weeds. In this study, we tested the resistance to three major herbicides, namely glufosinate, glyphosate and mesotrione of 421 diverse rice cultivars and identified 5 significant associations for rice resistance to glufosinate and glyphosate. By candidate gene-based GWAS, we predicted two genes, *LOC_Os06g39070* and *LOC_Os08g14850* for rice resistance to glufosinate and glyphosate, and found superior alleles for these two genes against glufosinate and glyphosate, respectively. Our results will shed light on the genetic diversity of rice resistance to herbicides and provide promising targets for enhancing rice resistance to herbicides.

## Data Availability

The datasets presented in this study can be found in online repositories. The names of the repository/repositories and accession number(s) can be found in the article/**Supplementary Material**.
