# Supplementary material for: Genome-wide association study reveals the genetic basis of rice resistance to three herbicides
Source: Front Plant Sci. 2024 Oct 1;15:1476829. doi: 10.3389/fpls.2024.1476829 (PMC11473433; doi:10.3389/fpls.2024.1476829)
Supplement: Supplementary file 1 [file DataSheet1.docx]

Supplementary Material

**Supplementary Material**

**1. Supplementary Figures**

**
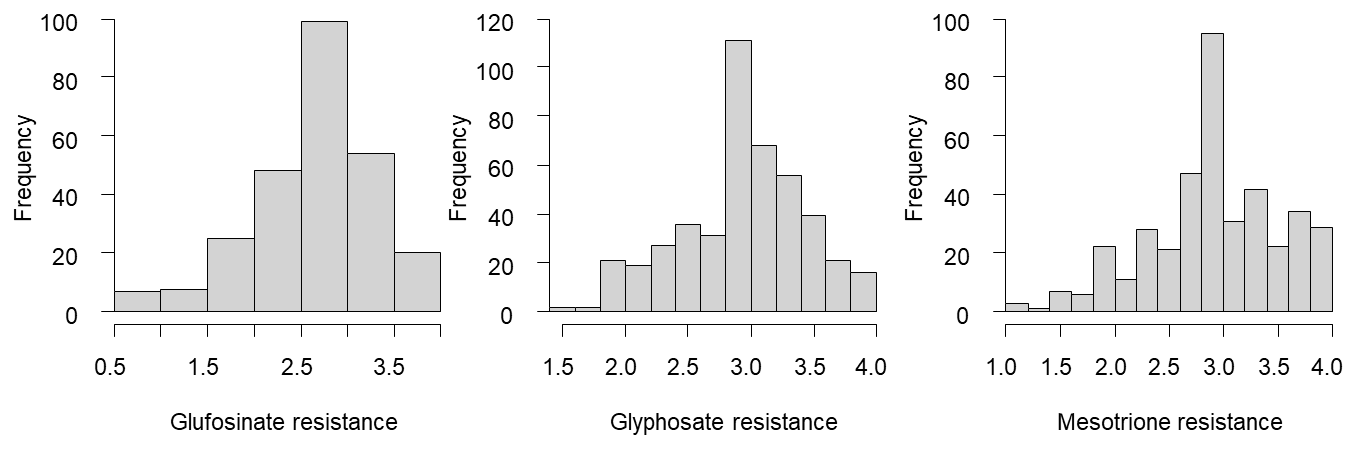
**

**Supplementary Figure 1.** Phenotypic distribution of three herbicides resistance in 421 diverse cultivated rice.

**
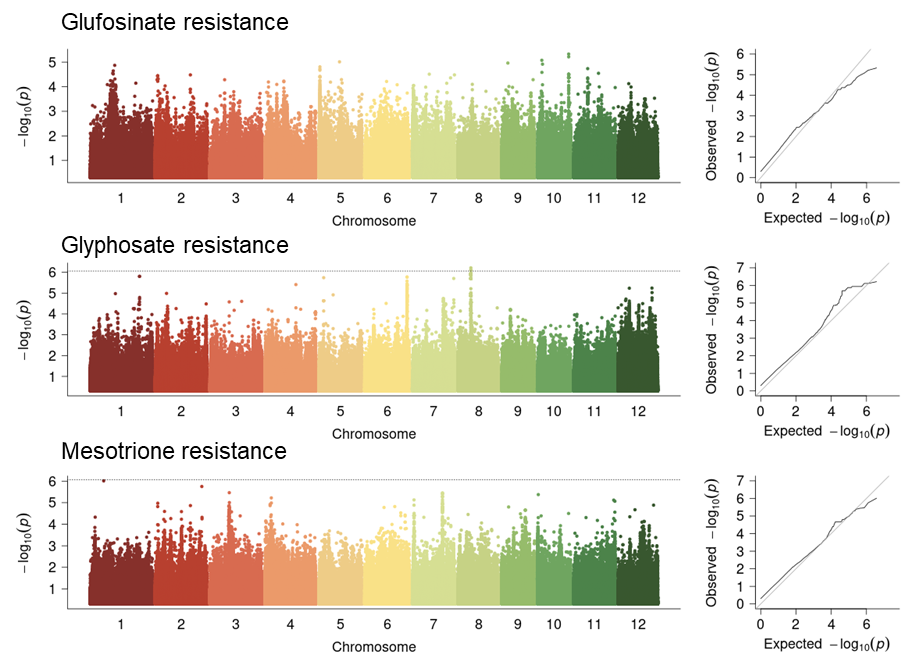
**

**Supplementary Figure 2.** Genome-wide association results for resistance to three herbicides in 300 *indica* rice varieties. Manhattan plots and quantile–quantile plots depicting GWAS results using a mixed model for resistance to glufosinate (a), glyphosate (b) and mesotrione (c). Associations identified in all accessions (lower), indica subspecies (middle), and japonica subspecies (upper). The x axis depicts the physical location of SNPs across the 12 chromosomes of rice and the y axis depicts the -log_10_(*P* value).

**
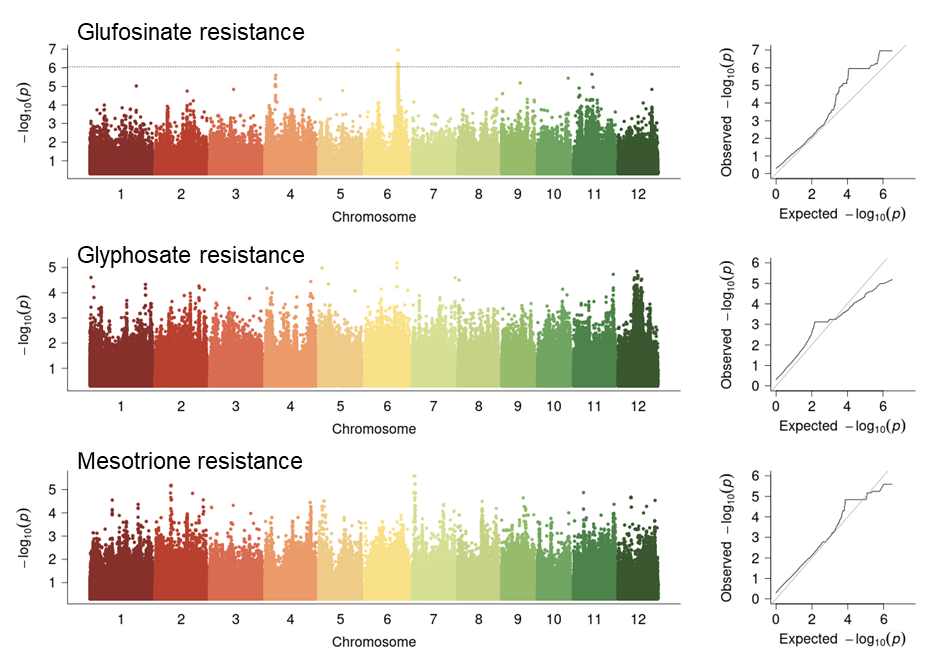
**

**Supplementary Figure 3.** Genome-wide association results for resistance to three herbicides in 106 *japonica* rice varieties. Manhattan plots and quantile–quantile plots depicting GWAS results using a mixed model for resistance to glufosinate (a), glyphosate (b) and mesotrione (c). Associations identified in all accessions (lower), indica subspecies (middle), and japonica subspecies (upper). The x axis depicts the physical location of SNPs across the 12 chromosomes of rice and the y axis depicts the -log_10_(*P* value).

**2. Supplementary Tables**

**Supplementary Table 1.** Estimated effective number of SNPs and significant thresholds in populations

| Population | Observed SNPs | Me | Effective ratio | Suggestive *P* value | Significant *P* value |
| --- | --- | --- | --- | --- | --- |
| All | 6,348,504 | 736566 | 0.12 | 1.41E-06 | 6.66E-08 |
| Indica | 3,878,260 | 560731 | 0.14 | 1.86E-06 | 8.89E-08 |
| Japonica | 1,968,956 | 234236 | 0.12 | 5.11E-06 | 2.13E-07 |

**Supplementary Table 2.** Phenotypic variation of glufosinate resistance explained by multiple QTL.

|  | *P*-value | | | *R^2^* |
| --- | --- | --- | --- | --- |
| *RGlu11* | 1.99×10^-12 |  |  | 17.43% |
| *RGlu6* |  | 1.44×10^-13 |  | 19.20% |
| *RGlu4* |  |  | 2.23×10^-11 | 15.95% |
| *RGlu11*+*RGlu6* | 1.61×10^-4 | 8.65×10^-6 |  | 23.65% |
| *RGlu11*+*RGlu4* | 9.29×10^-5 |  | 3.09×10^-3 | 20.21% |
| *RGlu6*+*RGlu4* |  | 4.3×10^-6 | 1.02×10^-3 | 22.71% |
| *RGlu11*+*RGlu6*+*RGlu4* | 1.15×10^-2 | 4.59×10^-4 | 0.07 | 25.76% |

**Supplementary Table 3.** Phenotypic variation of glyphosate resistance explained by multiple QTL.

|  | *P*-value | | *R^2^* |
| --- | --- | --- | --- |
| *RGly12* | 1.16×10^-6 |  | 5.15% |
| *RGly8* |  | 1.71×10^-20 | 20.06% |
| *RGly12+RGly8* | 8.28×10^-9 | 5.17×10^-23 | 26.69% |

**Supplementary Table 4. Information about the cultivars carrying the favorable *RGlu6* allele**

| Cultivar | Subpopulation | Glufosinate resistance | Area | Latitude | Longitude | vg0623197432 |
| --- | --- | --- | --- | --- | --- | --- |
| M202 | *Temperate Japonica* | 2 | India-Nepal | 28.6 | 77.2 | G |
| Longhuamaohu | *Temperate Japonica* | 2 | North China | 38.02 | 114.28 | G |
| Zaoshunonghu6 | *Temperate Japonica* | 2.5 | South China | 28.11 | 113 | G |
| Guihuahuang | *Temperate Japonica* | 3 | South China | 32.02 | 118.5 | G |
| Babaili | *Temperate Japonica* | 2.5 | South China | 25.04 | 102.41 | G |
| Ximaxian | *Temperate Japonica* | 1 | South China | 25.04 | 102.41 | G |
| Lengshuigu | *Temperate Japonica* | 1.5 | South China | 25.04 | 102.41 | G |
| Balilla | *Temperate Japonica* | 1 | Europe | 41.52 | 12.37 | G |
| Xingguo | *Temperate Japonica* | 1.5 | North China | 40.5 | 121.5 | G |
| Maguzi | *Temperate Japonica* | 1.5 | South China | 34.16 | 108.54 | G |
| Putaohuang | *Temperate Japonica* | 1.5 | North China | 39.09 | 117.11 | G |
| Sel._No._388 | *Temperate Japonica* | 2 | South America | -34.53 | -56.09 | G |
| KRASNODARSKIJ_424 | *Temperate Japonica* | 0.5 | Europe | 55.45 | 37.37 | G |
| Pergonil_15 | *Temperate Japonica* | 0.5 | Europe | 38.42 | -9.05 | G |
| NORIN_11 | *Temperate Japonica* | 1.5 | Korea and Japan | 35.7 | 139.8 | G |
| KUBANETS_508 | *Temperate Japonica* | 1 | Europe | 55.45 | 37.37 | G |
| HB-6-2 | *Temperate Japonica* | 0.5 | Europe | 47.26 | 19.15 | G |
| Niwahutaw_Mochi | *Temperate Japonica* | 1 | Korea and Japan | 35.7 | 139.8 | G |
| Gazan | *Temperate Japonica* | 1.5 | Middle East | 34.5 | 69 | G |
| Celiaj | *Temperate Japonica* | 2.5 | Middle East | 40.22 | 49.53 | G |
| CHUNJIANGZAO_NO._1 | *Temperate Japonica* | 2 | North China | 39.9 | 116.4 | G |
| KRASNODARSKIJ_3352 | *Temperate Japonica* | 1 | Europe | 55.45 | 37.37 | G |
| Bombilla | *Temperate Japonica* | 1 | Europe | 40.26 | -3.42 | G |
| LUSITANO | *Temperate Japonica* | 0.5 | Europe | 38.42 | -9.05 | G |
| Yunguang8hao | *Temperate Japonica* | 1.5 | South China | 25.04 | 102.41 | G |
| M401 | *Temperate Japonica* | 0.5 | Philippines | 14.6 | 121 | G |
| Daegujo | *Temperate Japonica* | 1 | Korea and Japan | 37.35 | 127.03 | G |

**Supplementary Table 5. Information about the cultivars carrying the favorable *RGly8* allele**

| Cultivar | Subpopulation | Glyphosate resistance | Area | Latitude | Longitude | vg0808951557 |
| --- | --- | --- | --- | --- | --- | --- |
| AnnongwangengB-2 | *Temperate Japonica* | 2.00 | South China | 30.14 | 120.09 | C |
| Funingzipi | *Temperate Japonica* | 2.00 | North China | 38.02 | 114.28 | C |
| Longhuamaohu | *Temperate Japonica* | 2.17 | North China | 38.02 | 114.28 | C |
| Gaoyangdiandao | *Temperate Japonica* | 2.50 | North China | 38.02 | 114.28 | C |
| Lixingeng | *Temperate Japonica* | 2.33 | South China | 30.39 | 104.05 | C |
| Tieganwu | *Temperate Japonica* | 2.33 | South China | 30.14 | 120.09 | C |
| Balilla | *Temperate Japonica* | 1.83 | Europe | 41.52 | 12.37 | C |
| Heigeng2hao | *Temperate Japonica* | 2.00 | North China | 45.45 | 126.41 | C |
| Feidongtangdao | *Temperate Japonica* | 2.00 | South China | 37.51 | 117.18 | C |
| Putaohuang | *Temperate Japonica* | 2.50 | North China | 39.09 | 117.11 | C |
| Sel._No._388 | *Temperate Japonica* | 2.50 | South America | -34.53 | -56.09 | C |
| KRASNODARSKIJ_424 | *Temperate Japonica* | 2.50 | Europe | 55.45 | 37.37 | C |
| Pergonil_15 | *Temperate Japonica* | 2.83 | Europe | 38.42 | -9.05 | C |
| Chacareiro_Uruguay | *Temperate Japonica* | 3.33 | South America | -34.53 | -56.09 | C |
| KUBANETS_508 | *Temperate Japonica* | 1.50 | Europe | 55.45 | 37.37 | C |
| HB-6-2 | *Temperate Japonica* | 1.50 | Europe | 47.26 | 19.15 | C |
| Tamanishiki | *Temperate Japonica* | 2.00 | Korea and Japan | 35.7 | 139.8 | C |
| Somewake | *Temperate Japonica* | 2.00 | Korea and Japan | 35.7 | 139.8 | C |
| Vary_Tarva_Osla | *Tropical Japonica* | 2.00 | Europe | 38.42 | -9.05 | C |
| CSORNUJ | *Temperate Japonica* | 2.33 | Europe | 47.26 | 19.15 | C |
| Celiaj | *Temperate Japonica* | 2.00 | Middle East | 40.22 | 49.53 | C |
| WIR_911 | *Temperate Japonica* | 1.50 | Europe | 55.45 | 37.37 | C |
| KRASNODARSKIJ_3352 | *Temperate Japonica* | 2.50 | Europe | 55.45 | 37.37 | C |
| Bombilla | *Temperate Japonica* | 2.00 | Europe | 40.26 | -3.42 | C |
| LUSITANO | *Temperate Japonica* | 3.17 | Europe | 38.42 | -9.05 | C |
| M401 | *Temperate Japonica* | 3.33 | Philippines | 14.6 | 121 | C |
| Teksichut | *Temperate Japonica* | 2.50 | Philippines | 14.6 | 121 | C |
| Eiko | *Temperate Japonica* | 3.67 | Korea and Japan | 35.7 | 139.8 | C |
